# Experimental Cholecystotomy

**Published:** 1884-09

**Authors:** J. McF. Gaston

**Affiliations:** Atlanta, Ga.


### EXPERIMENTAL CHOLECYSTOTOMY.

BY J. McF. GASTON, M. D., ATLANTA, GA.

With a view to demonstrate the practicability of uniting the walls of the gall-bladder and upper part of the smaller intestine by an elastic ligature and effecting a communication between them, while the stitches encircling this shall secure adhesion of their adjacent surfaces, I have undertaken this operation upon dogs.

These experiments have been made on several dogs and were witnessed at various times, in one or more cases, by eleven different colleagues in this city.

August 9th, 1884.—A young dog, Case No. 1., was operated on under the anæsthetic influence of sulphuric ether, by making an oblique incision from the median line, about one inch below the ensiform cartilage and extending at the same distance from the margins of the false ribs for six inches on the right side. The gall-bladder being deeply situated beneath the anterior lobe of the liver, was reached with some difficulty by one assistant holding the liver up and at the same time drawing it out, while another pushed his fingers downward upon the stomach and pancreas with the meso colon. If this were a proper occasion for noting points of comparative anatomy, the bluish hue of the gall-bladder as contrasted with the greenish yellow of the human race, is worthy of our attention, and the location of this receptacle being in the dog more deeply than in man, under the anterior margin of whose right lobe it is found with its pear shape differing from the somewhat differently formed gall-bladder of the dog.

These points may guide others in repeating my experiments, and yet it may be observed that the location varies considerably in different dogs, so that the difficulty encountered in this case was not found in others. Having brought that portion of the duodenal canal immediately below the entrance of the ductus choledochus into close proximity with the gall-bladder, a needle having the shape of a fishhook and armed with an elastic ligature, was passed through the most salient portion of the wall of the gall-bladder so as to include nearly half an inch of surface and then carried through the wall of the intestine so as to include about half an inch of its surface. The ligature, with these tissues in its loop. was tightened so as to embrace both walls closely and knotted. A fine needle with catgut ligature was used to attach the surrounding serous surfaces by a circular line of continuous stitching, with the hope of securing their union by adhesive inflammation. Thus it is expected that when the elastic ligature shall cut its way through the approximated walls of the gall-bladder and the intestinal canal, so as to afford a communication from the former into the latter, there shall be formed adhesions around this fistulous opening by which any escape of the contents of either into the peritoneum shall be obviated. The internal facing of the abdominal wound was closed with continuous sutures of catgut, while the external was united by interrupted sutures of silk. This dog having been kept under the influence of the sulphuric ether for one hour, it was quite a considerable time before the effects of it passed off, and in subsequent experiments this observation was utilized by suspending the inhalations so soon as the principal steps were completed.

This animal took beef soup thickened with corn meal during the following day, and drank water in moderation, so that on the morning of the 11th all seemed to be progressing well, not only locally but as to the general condition.

August 11th, 1884.—Two dogs, cases No. 2 and 3, were operated on as the other, with the use of ether as an anæsthetic. The external incision being carried a little to the left of the mesial line in view of the observation made as to the anterior location of the gall-bladder, it was not extended obliquely backward more than five inches.

The experiment was varied in these two cases from the course pursued in the first case by attaching the wall of the gall-bladder to the duodenum above the duct instead of to the canal below its insertion, as thus tension was lessened upon the delicate tissue of the sac, but not with any conviction of its advantages in approximating the normal functions of the bile in completing digestion. There is besides such an increase of thickness in the wall of the duodenum above the insertion of the duct that the latter part will be more likely to yield within the same period which is requisite for the wall of the gall-bladder to give way to the constriction of the elastic ligature. In view of these facts this plan of union was adopted in the subsequent experiments.

August 12th, 1884.—The dog operated upon on the 9th instant seems to be doing well, and the other two subjects of yesterday's experiments are in good condition. All have a disposition to take food. Another dog was etherized to-day, and the external cut was made as in the latter two. from the left of mesial line diagonally downwards to the right, but extending somewhat less than five inches, as it was intended to simplify the operation in this case, No. 4.

Unluckily the meso-colon was caught over the point of the directory and wounded in making the peritoneal incision so as to require ligation of a small artery.

Being desirous of finding out whether the margins of the walls united by the elastic ligature would take on adhesive inflammation without the circular stitching around it, I only passed the ligature through the gall bladder and the intestine immediately below the orifice of the duct, drawing the two surfaces together, where it was insecurely knotted and left for subsequent observation of the result, as one end of the ligature broke off in tying it.

The interior peritoneal margins were closed with continuous catgut sutures, and with a view to vary the experiment a coarser thread of catgut, with a larger needle, served to unite the external incision by a continuous suture.

August 13th, 1884.—Case No. 5 of the canine race was placed under the influence of ether, and the external incision made as in the subject of yesterday's experiment. The elastic rubber ligature being passed with the fish-hook shaped needle through the walls of the gall bladder and the upper small intestine below the entrance of the common bile duct, was securely knotted, so as to retain their exterior surface in close apposition.

A fine needle with a catgut suture was used to bring the tissues of the gall-bladder and the duodenum together in a circle around the ligature and gives promise of securing adhesion between them.

As in the other cases the peritoneal incision was closed by continuous catgut sutures, and the exterior wound was stitched with interrupted sutures of silk After the muzzle was removed and the dog placed in his cage it was observed that the effects of the ether had passed off, as there had been no inhalation of it while sewing up the abdominal wall.

Upon examining the state of the wounds in the subjects of the previous experiments, it was found that in No. 2 and 3 several stitches had given away or been torn loose by their teeth, so that it was requisite to replace them.

In No. 1 there was but a single stitch of the interrupted suture lacking at the posterior extremity of the incision, and as there was but little gaping, it was not thought necessary to replace it. As was noticed in my account of the operation in this case, considerable traction had been made upon the right lobe of the liver with more handling of the viscera than could be well borne, and supposing there might be inflammation with some suppuration, this opening was left for the escape of any purulent discharge.

No antiseptic applications were made in any of the cases either during the operations or subsequently, except that the catgut had been put up in carbolized oil, and some pieces seemed to be impaired by it. One ligature was also brittle after use.

Yet upon immersing a piece of the elastic which had been smeared with bile and gastric juice from the ox, the ether caused no alteration in the texture, so that I am still without a clue to the marked change observed in the pieces of ligature which remained from the operation on the dog to-day.

August 14th, 1884.—A survey of my canine infirmary gave the sad spectacle in Case No. 3 of a large portion of the small intestines protruding from the wound and covered with dirt from dragging on the ground. After washing them thoroughly in cold water with the hope to reduce somewhat the engorgement and evident peritoneal inflammation, they were returned to the abdomen and the wound closed by interrupted sutures, including the internal coating with the skin. A broad band of cloth was then sewed around the body of the animal so as to support the parts and prevent contact of its mouth; but the indications of sphacelus in the margins led to the conclusion that death was near.

Case No. 4, in which the wound had been closed with continuous coarse catgut sutures, was found to be gaping slightly at some points, and a few stitches of interrupted suture were inserted to effect complete co-aptation of the margins.

Case No. 1 lies stretched out at full length on the ground, as if there was no prospect of living much longer.

August 15th, 1884.—Early this morning Case No. 3 was found stiff and cold, having succumbed during the night.

Upon opening the wound all the evidences of peritonitis in an intense form were presented with slight effusion in the abdominal cavity. The anterior extremity of the incision was carried upwards on the left side of the ensiform cartilage so as to expose the region of the liver, and after drawing out the small intestines the duodenum was found agglutinated by recent adhesion to the lower surface of the liver.

At this point the attention of my colleage, Dr. K. C. Divine, was called to the state of the parts and we concurred in the impression that there was less evidence of acute inflammation in the duodenum and the adjoining portion of the pancreas than in the folds of the small intestine. Detaching the slight adhesions of the serous surfaces of the duodenum and liver, the attachment of the gall-bladder by the fine catgut to the wall of the duodenum was still preserved so that the only mode of reaching the site of the elastic ligature within this circular stitching was by cutting open the canal of the duodenum until the point was reached in which the ligature had been made. It was discovered that the opening into the gall-bladder had been effected, and that the elastic ligature had disappeared, doubtless in the intestinal tube.

Wishing to preserve the specimen, a dissection of the surrounding substance of the liver was made, but in drawing it out rather too forcibly, the weak attachment of the gall-bladder and duodenum was broken up so that my pathological case only illustrates the practicability of effecting a communication with surrounding adhesions of the serous surfaces.

It will be understood that the gall-bladder was entirely empty and flaccid, as the aperture in its wall and that of the duodenum gave a direct outlet to the bile.

One important observation was made in this result, that the opening was much larger than requisite or desirable; and in repeating this experiment a much smaller portion of the tissues should be included in the loop of the ligature. It would, perhaps, suffice to pass the needle only to the extent of one-fourth of an inch through each surface, but reaching the cavity with certainty in either case, so that the ligature shall effect a communication between the gall-bladder and the alimentary canal, which, as I have already stated, should be ligated in its wall below the entrance of the bile duct.

There is satisfactory evidence in the prompt action of the elastic ligature within less than four days, so that my fears in regard to the loss of elasticity and strength by contact with the secretions were unfounded, and yet perhaps it would be better to use a slower cutting ligature.

August 16th, 1884.—Case No. 1. contrary to my expectations, is still alive this morning, and was found sitting up upon his haunches, looking around him with a much more encouraging aspect; and though the opening at the lower end of the incision remains, it was not thought requisite to do more than sew a cotton cloth band around the body in this case as I did in all the others. Nos. 4 and 5 had licked their wounds so as to detach several points of the suture, and the continuous suture used in the former no longer seems to keep the edges united. It was, therefore, necessary to insert several stitches of the interrupted suture in each of these cases, and subsequently cover the wounds by a compress and bandage with a view to prevent if possible any further trouble from their mouths. The belly-band was placed also upon case No. 2, though it has continued throughout in better condition than the others as to the stitches in the incision.

August 17th, 1884—The four subjects of the experiments are all alive still, and yet case No. 4 presented a small protrusion of omentum in the incision, where one of the stitches had given way. There were also other points of the interrupted suture torn loose, which required to be replaced, but the tissues within the margins gave no indications of sloughing, so that the sutures were most probably detached by the mouth of the animal. The bandage placed around each of the dogs yesterday morning was entirely useless, by rolling back in a cord around the loins or reins of the animal. Case No. 2 got loose on yesterday, and it was reported to me by the person in charge that there was scarcely any evidence of disability in the movements of the animal. The wound in this case still presents a good appearance, as likewise that of case No. 5. Case No. 1 is lying extended upon the ground this morning and the hopes inspired on yesterday are reduced to the minimum of expectancy.

August 18th, 1884.—No. 1 seems to have a new lease on life this morning, and moves about to the extent of the tether, presenting signs of agglutination in the borders of the incision. No. 2 bids fair to escape a post mortem exploration and afford me an opportunity of testing the effect of ligation of the bile duct after the communication is fully established from the gall-bladder into the duodenum. This plan of occlusion subsequent to securing a direct opening has the advantage over a primary ligation, that a free flow of the bile relieves this case of those serious troubles which accompany obstruction, and are known as hepatic colic. In chronic disorders of the biliary apparatus in the human subject, the interruption of this secretion or excretion, as it may be considered, having come about gradually, and led to structural changes in the gall bladder, it is evident that the connection of some part that is most dense with the wall of the duodenum is likely to give the best result.

No. 3 has already afforded very satisfactory indications by a post mortem exploration.

No. 4, in which it will be recollected I simply passed the elastic ligatures through the walls of the gall-bladder and the canal, is found to-day again with protrusion of the omentum from the stitches being torn out of the part of the incision nearest the mesial line. After washing and returning the omentum several points of interrupted sutures were required to close the wound.

No. 5 presents a favorable aspect, excepting the very annoying feature of having to replace several stitches that were torn loose.

August 18th, 1884, Afternoon.—Receiving notice of the death in case No. 4, I proceeded, at 3 o'clock, p. m., to make a post mortem examination. The external wound was accurately closed, as it had been left in the morning, and the stitches being cut, the first thing that presented at the opening was a coil of small intestine which gave evidence of a high state of peritoneal inflammation. Carrying the incision anteriorly upward along the right side of the ensiform cartilage, and posteriorly between the eighth and ninth ribs, the liver and adjacent tissues were dissected out, cutting across the pyloric orifice of the stomach. It was apparent that the gall-bladder was full, and hence clear that my experiment was an entire failure, and upon inspecting the adjacent portion of the duodenum the piece of elastic ligature unknotted was lying loosely upon its external surface with very marked indications of thickening in the intestinal wall and a very minute opening into the cavity. Upon a close examination of the gall-bladder its surface was found adhering at a small circumscribed spot to the under side of the liver, and upon breaking up this union the cicatrix of the wound made by the ligature was discovered giving indications that the wall had been cut through partially. Nature had done good surgery in soldering the opening, and indeed there was nothing in the state of the parts to have caused a fatal result but for the peritonitis, set up doubtless by the protrusions of the omentum two days in succession.

The unknotting of the elastic ligature is explained by the fact of the break occurring on the occasion when it was applied; and when the tension was relieved by partially cutting through the tissues of the gall-bladder and the duodenum it was untied by its own elasticity. The substance of the elastic strip had undergone no change, and upon drawing it out by seizing each extremity, the strength was sufficient to resist considerable tension and yet not break.

It will naturally occur to those who read these details that measures should have been adopted from the outset to prevent the contact of the dogs' mouths with the incisions, but in attempting to keep them muzzled they kept up an incessant effort with their fore feet until it was removed. If the head was fastened close to a post or wall, it caused a state of restlessness that was not favorable to a good result. The only proceeding which is likely to be advantageous in these cases is suspension by a band beneath the abdomen, extending entirely from the fore shoulders to the quarters, with another support around the head and mouth so as to keep the animal from using either the mouth or the paws. I will put this into execution with these subjects now, and in future it will be resorted to immediately upon closing up the incisions in abdominal experiments.

It was thought that some licking of the wounds might keep them clean, and that the freedom of the dog would be a security against being fly-blown, but the serious inconvenience in other respects convinces me of the absolute necessity of adopting the above precaution against any kind of disturbance to the sutures.

As this report cannot be extended further in view of the demand of the printer, other details of the progressive developments in the living or dead will be printed in the next number of THE JOURNAL.

